# Identifying and Analyzing Dependencies in and among Complex Cyber Physical Systems

**DOI:** 10.3390/s21051685

**Published:** 2021-03-01

**Authors:** Aida Akbarzadeh, Sokratis Katsikas

**Affiliations:** Department of Information Security and Communication Technology, Norwegian University of Science and Technology, N-2815 Gjøvik, Norway; sokratis.katsikas@ntnu.no

**Keywords:** cyber physical systems, system of systems, graph theory, multi-order dependencies, cybersecurity

## Abstract

Contemporary Critical Infrastructures (CIs), such as the power grid, comprise cyber physical systems that are tightly coupled, to form a complex system of interconnected components with interacting dependencies. Modelling methodologies have been suggested as proper tools to provide better insight into the dependencies and behavioural characteristics of these complex systems. In order to facilitate the study of interconnections in and among critical infrastructures, and to provide a clear view of the interdependencies among their cyber and physical components, this paper proposes a novel method, based on a graphical model called Modified Dependency Structure Matrix (MDSM). The MDSM provides a compact perspective of both inter-dependency and intra-dependency between subsystems of one complex system or two distinct systems. Additionally, we propose four parameters that allow the quantitative assessment of the characteristics of dependencies, including multi-order dependencies in large scale CIs. We illustrate the workings of the proposed method by applying it to a micro-distribution network based on the G2ELAB 14-Bus model. The results provide valuable insight into the dependencies among the network components and substantiate the applicability of the proposed method for analyzing large scale cyber physical systems.

## 1. Introduction

With the rapid growth of merging Information and Communication Technology (ICT) with Critical Infrastructures (CIs) such as Energy and Transportation systems, the complexity of CIs drastically increased and Cyber–Physical Systems (CPSs) have been formed. CPSs are the result of integrating the computing, communication, and control capabilities, with physical processes which were developed to facilitate the monitoring and controlling of system components in the physical world [1]. Although this progress enhanced the efficiency and service coverage of CIs, it significantly increased the connections among the system components as well as the interdependencies between different sectors of CIs, such as the dependency between the transportation system and the power and telecommunication systems.

These intricate dependencies make systems more vulnerable because in this way any failure of critical infrastructure will have a considerable impact not only on the infrastructure itself but also on the other dependent infrastructures. As an example, in 2001 an electric power disruption in California, caused a cascading failure and affected oil and natural gas production, refinery operations, gasoline transportation, key industries and the water and agriculture sectors, which led to major financial loss [2]. Two years later the blackouts in the USA–Canada and Southern Sweden and Eastern Denmark revealed the possibility of international cascading effects. In general, recent blackouts [3] and studies on their impact [4] clearly showed this strong dependency between the electrical infrastructure as an individual CPS and other CIs and the consequences of this dependency.

Meanwhile, the frequency and impact of recent blackouts, particularly in Europe and North America, are progressively growing; this could also be interpreted as a remarkable warning for all CIs [5,6]. The vulnerability of the electrical infrastructure by itself mainly stems from the heterogeneity of connections and dependencies among the system components. This vulnerability has grown after the merging of the electrical infrastructure with information and communication systems and turned electrical infrastructure into an attractive target for cyber attacks. In electrical infrastructure, like other CIs, any individual part and facet of a system has its special characteristics; this affects the behaviour of the entire system when it encounters an unexpected situation such as a cyber or a cyber physical attack [7]. Therefore, detecting the chain of dependency and studying the relationships among components of CIs, particularly inside the electrical infrastructure as vital cyber physical systems, are of great importance for the maintenance of key processes which substantially impact on the economy and societal well being.

Modelling and simulation methods are highly suggested as proper tools to study CPSs. With the main goal of enhancing the resilience and security of complex systems, valuable researches have been conducted for modelling the dependencies of and in such systems; these include Complex Networks Theory/Graph Theory, Petri-Nets [8], Well-Formed Nets (SWN) [9], Input-Output Models [10], Bayesian Networks [11], Matrix representations, Boolean logic Driven Markov Processes (BDMP), Agent-Based Models and Multi-Agent Modelling [12]. Most of the aforementioned studies focus on qualitative or semi-qualitative analyses. Unfortunately, such approaches provide inadequate knowledge to system designers and decision-makers with the responsibility to mitigate negative impacts and to manage risks arising from dependencies inside a system, since operators not only need to know about the connectivity and dependencies, but their magnitude and characteristics as well [13,14].

Despite significant efforts in recent years, analysis and modelling of CPSs is still a challenging problem in basic research on complex systems; because in this context, CPSs are not analysed as discrete assets or services within particular sectors. Instead, a holistic system-of-systems view is followed, in which all the connections between different subsystems and sub-layers of a CPS are considered [15]. Even though Graph Theory-based methods were known as the most common and effective approaches to reveal the hidden dependencies [16], reviews of recent studies show that utilizing Graph Theory to study large scale systems such as electrical infrastructures will result in massive complicated diagrams that cannot be easily understood and cannot assist in distinguishing the impact of dependencies [17]. Nevertheless, graphical models developed based on Graph Theory such as Network Analysis and Design Structure Matrix (DSM) have addressed these issues to some extent and represented promising results to evaluate the characteristics of connections in CPSs. DSM has been mainly developed to extract the interrelationships exist between the activities of a complex design problem to break them down into smaller sub-problems. More precisely, in the DSM, the connectivity between the elements of a system should be represented in the form of a matrix first and then different methods such as clustering will be applied to find probable dependencies or structural patterns that might exist. However, due to the fact that this model requires to analyse of all the system connections to extract probable dependencies, DSM could not be an efficient method to study characteristics of connections in large scale CPSs. To tackle this challenge, we propose MDSM, a modified version of DSM in which the searching based algorithms in the analysis phase of the DSM are replaced with a lightweight and deterministic approach. Indeed, MDSM not only has lower computational complexity but also extracts the characteristics of connections for all the system components and represent the result in a predefined systematic structure, unlike DSM. Moreover, to facilitate the quantitative analysis of dependencies of complex systems, the inter-dependency and the intra-dependency are located in predefined and separate parts in the MDSM.

Indeed, applying a graphical model to represent the interconnections between different subsystems of a large-scale CPS effectively enhances the knowledge about the connectivity within the systems and presents more details on the behaviour of different subsystems while working as a whole, in particular on their interdependencies. Therefore, this paper first attempts to develop a simple yet useful graphical method to represent coupled critical infrastructures to facilitate the identification of dependencies within CIs and then proposes quantitative parameters to evaluate the characteristics of dependencies inside large scale systems in order to enhance the security and robustness. Our main contributions are as follows:

- We propose MDSM as a graphical model to extract characteristics of connections inside a cyber-physical system to facilitate studying the behaviour of dependent components of large scale systems including, both intra-dependency and inter-dependency.
- We propose four quantitative dependency parameters, namely the Impact of Dependency (IoD), the Susceptibility of Dependency (SoD), the Weight of Dependency (WoD) and the Criticality of Dependency (CoD) to measure the characteristics of dependencies.
- We propose a method to aggregate quantitative dependency parameters of the higher order of dependency to evaluate the characteristics of multi-order dependencies in CPSs.
- We illustrate the application of the proposed method to a reduced scale network from a real French Distribution Network with 14 power-bus.

The rest of the paper is organized as follows: In Section 2, we review the related work on modelling dependencies in CIs. Section 3 describes the proposed method, while Section 4 explains the concept of the higher order of dependency in system-of-systems. A case study is presented in Section 5 to evaluate the applicability of the proposed method and application of dependency analysis is expounded in Section 6. Finally, Section 7 summarizes our conclusions and indicates directions for future work.

## 2. Related Work

As discussed earlier, critical infrastructures depend on each other to operate properly and these expanding connections among them, be they tangible or intangible, have increased the vulnerabilities of CIs. The term dependency refers to a connection or linkage between two components, through which the state of one component influences the state of the other. While interdependency is a two-way dependency, a mutual dependency, between two components such that the state of each component influences or is correlated to the state of the other one.

Exploiting the six dimensions of interdependencies proposed by Rinaldi et al. [2] namely, type of failure, infrastructure characteristics, state of operation, environment, coupling and response behaviour and types of interdependencies, facilitates the identification of interdependencies inside CIs. Each dependency between two components may be represented by modelling the connection between them, which is one of the following types:

- Input, Mutual, Shared, Exclusive, Co-located [18];
- Physical, Cyber, Geographic, Logical [2];
- Functional, Physical, Budgetary, Market and economic [19];
- Physical, Geospatial, Policy, Informational [20].

Ouyang et al. [21] developed ten different scenarios to evaluate these types of dependencies in CIs and concluded that utilising the type of interdependencies proposed by [2] provides better results in terms of covering a variety of scenarios. Nieuwenhuijs et al. [22] asserted that the geographical interdependencies are the result of a common mode failure rather than a type of dependency that was mentioned in [2]. Rinaldi et al. [2] proposed Cascading failure, Escalating failure and Common cause failure as three different types of dependency-related failures as a dimension of dependency. Later, the result of an empirical study indicated that dependency-related failures in systems could be categorized into either cascade-initiating or cascade-resulting [23]. In general, analysing dependencies through this dimension increases the system resilience as it facilitates the identification of failures that might occur in CIs. Such failures can disturb the functionality of systems, thus affecting their reliability. Modelling dependencies of CIs in order to understand the behaviour of complex systems encountered with failures that may be caused by adversaries is a common approach towards enhancing the reliability of systems [24,25]. In general, modelling CIs in terms of their interdependencies provides an insightful view of inter-system and intra-system causal relationships, response behaviour, failure types, state of operation, and risks that arise due to the dependency-related failures in systems [26,27]. Accordingly, significant efforts have been made to develop appropriate models to map out the interdependencies of complex systems. Even though several researchers attempted to model dependencies between all the critical infrastructures [28,29,30], the majority focused on limited numbers of critical infrastructures [31,32], particularly on the power and ICT infrastructures [33,34].

In fact, large scale blackouts and the ongoing transition towards smart grids and the idea of developing smart cities across the globe decisively highlighted the impact of the power systems on the reliability of all CIs in different sectors [35]. An empirical study on different CIs showed that energy and telecommunications are the main cascading-initiating sectors [23]. As a result, significant efforts have been made in the last few years to study and model the interdependencies of power systems combined with ICT systems, viewed as complex cyber-physical systems, to improve defensive and protective strategies in the cyber and physical layers of power systems [33,34,35,36,37].

Researchers in many domains attempt to identify suitable methods to model real systems, considering the relations and dependencies between the systems’ components. Satumtira et al. [38] surveyed 162 papers on interdependency modelling, among which the Graph Theory/Complex Network Theory (at 22% of the studies) was the most common method to study interdependencies in CIs. Input-output models were next, followed by agent-based models that were used in 11% of the studies. Each of these methods has its own advantages and weaknesses in modelling CIs in terms of different dimensions of interdependency. For instance, the input-output model, that is inherently a method to study the economic flow, has been applied recently to calculate economic losses that result from the unavailability of different sectors in CIs and their interdependencies. This model has also been modified in a way that could evaluate the spread of risk among system components [39,40]. Nevertheless, input-output modelling may not be used in holistic approaches to capture both functional and geographic interdependencies [41].

Torres [42] suggested six different objectives namely Scalability, CPU time, Usability, Tools accessibility, Dynamic simulation and Large systems modeling to evaluate different methods including Agent-based Model, Petri Nets, Bayesian Networks, BDMP and Complex Network Theory/Graph Theory for modelling CIs. Comparing those methods by the author revealed that the Complex Network Theory with the highest value in four out of six different objectives has the best results, which confirmed the applicability of this method to model CIs [42]. Indeed, the Complex Network Theory is developed based on the Graph Theory to study real networks in social and computer science, biology, telecommunication, transport, electronics, electrical engineering, and other domains with complex systems [43].

According to Graph Theory, topological analysis allows us to describe the connectivity of complex systems and to model the relationships between system components and their characteristics with less data. The topology-based method facilitates vulnerability assessment and can provide a clear view of the role and importance of each component and connection in the systems, as well as to fully cover all types of interdependencies; no other model has this ability [21,44,45]. Therefore, this method is a suitable choice for analysing complex systems, since it explicitly includes the interactions and dependencies within/between systems and provides a simple yet powerful means to evaluate and manage complex systems architectures [35].

Likewise, derivatives of Graph Theory in the context of the topological analysis, such as matrix-based system modelling representation (Adjacency matrix) and Network Analysis and Design Structure Matrix (DSM) visualize the system components and interactions as graphical nodes and lines [46,47]. This intuitive model reduces the complexity of the analysis process and contributes to improving the understanding of operators [48]. DSM is known as a highly flexible and straightforward modelling technique, which provides valuable insights for engineers and managers in a wide range of fields. This method was initially developed to decompose a complex design problem into sub-problems by displaying the interrelationships between the activities in the form of a matrix. Recently, DSM has been utilized in different fields to study interdependencies; as a result, it is currently referred to as Dependency Structure Matrix Analysis [49]. Eppinger et al. presented the application of DSM in different industries and sectors through 44 practical cases [50]. The growing dependency-related failures within CIs, and the significant impact of CIs on the economy and the quality of life, intensify the necessity of developing modelling methods to study the dependencies and characteristics of complex systems, in particular, for modelling large scale CIs such as power and ICT systems.

**Figure 1:**
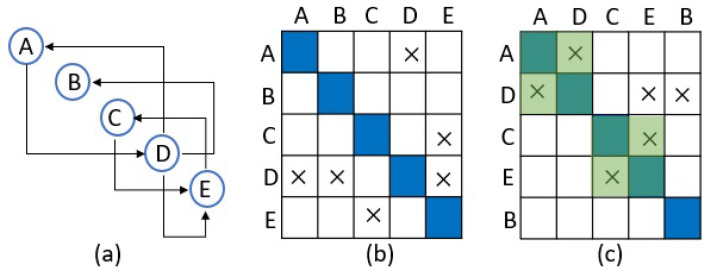
A sample digraph (**a**), its equivalent DSM (**b**) and the result of DSM sequencing which is indicated in green blocks (**c**).

DSM represents the interaction among the elements of a system in a square matrix with the inputs in rows and outputs in columns (Figure 1b). Then, based on the type of the system and its application, different analytical methods such as the clustering and sequencing analysis can be applied to extract the relations among the desired elements of the system (Figure 1c). In other words, DSM first documents the relationships among the elements of a system and then utilizes clustering analysis and rearranges the system’s elements in order to find structural patterns that might exist in the system, such as an interdependency.

We propose the modified DSM in Section 3 to turn the DSM into a predefined systematic structure for representing interactions between two subsystems without the need for those analytical methods. In this way, not only the computational complexity will decrease, but MDSM will also assist in extracting the characteristics of connections for all the system components, unlike the DSM. In MDSM the direction of connections between components is clearly distinguishable and inter-dependency and intra-dependency are placed in predefined and separate parts; this greatly facilitates further analysis and calculations. We also introduce four dependency parameters to evaluate and analyse the weight, impact and criticality of each dependency relationship between components in a quantitative manner.

## 3. Modified Dependency Structure Matrix (MDSM) Method

In this section, the process of forming an MDSM to representing the relationships between two subsystems in a CPS is described, and different characteristics of dependencies within a complex system are extracted from the MDSM. The applicability of the proposed method in large scale CPSs is also explored in more detail. The MDSM method is a graphical approach to demonstrate the dependencies and interdependencies between two subsystems of a CPS. The whole process follows a six-step approach, namely Set up, Modify, Rearrange, Display, Identify and Analyze. The outcome is represented as a square N×N matrix, in which *N* contains the elements of both subsystems. Each of these steps is described in the following:

### 3.1. Set Up (Step 1)

The first step of MDSM is to define two domains or subsystems of interest and capture the connections inside each subsystem, as well as between two subsystems. The collected data could simply be mapped as a directed graph *D* to show elements of each subsystem and their connections, with the direction being preserved. In Graph Theory, a directed graph or in short form digraph *D* is a pair (V,A) where *V* is a set of vertices (nodes) and *A* is a subset of V×V≡{(x,x)|x∈V} called arcs. If (u,v)∈A then the arc $a=\langle \vec{u,vs.}\rangle$ joins the initial vertex (tail) *u* to its terminal vertex (head) *v* [51].

Once the essential data are collected, we use the adjacency (or connectivity) matrix *A* of order *N*, where *N* denotes the total number of nodes, to represent the result. Rows and columns of matrix *A* are labeled according to the total number of elements while grouped into subsystems, and each row and its corresponding column in *A* is filled out taking into account the direction of the connection. *A* is a binary matrix and each nonzero value in row *i* column *j* indicates an arc $\langle \vec{i,j}\rangle$ which means that node *j* depends upon node *i*.

Without loss of generality and for the sake of clarity, suppose that we want to apply MDSM to study the dependency characteristics and connectivity properties of a smart grid system, a power-communication network that comprises both power and communication components, and that the two subsystems of interest are the Power system (Physical) and the Communication system (Cyber). Subsequently, first we need to collect the topological data of all nodes in the physical part $V_{p}\equiv \left\{v_{1},v_{2},\ldots ,v_{p}\right\}$, and the cyber part $V_{c}\equiv \left\{v_{1},v_{2},\ldots ,v_{c}\right\}$. Then, the matrix *A* of order N=(p+c) is set up to illustrate the relationship between each pair of nodes as follows:

$$A_{N,N}=\left(\begin{array}{cccccc} a_{p_{1}p_{1}} & \cdots & a_{p_{1}p_{p}} & a_{p_{1}c_{1}} & \cdots & a_{p_{1}c_{c}}\\ : & ... & : & : & ... & :\\ a_{p_{p}p_{1}} & \cdots & a_{p_{p}p_{p}} & a_{p_{p}c_{1}} & \cdots & a_{p_{p}c_{c}}\\ a_{c_{1}p_{1}} & \cdots & a_{c_{1}p_{p}} & a_{c_{1}c_{1}} & \cdots & a_{c_{1}c_{c}}\\ : & ... & : & : & ... & :\\ a_{c_{c}p_{1}} & \cdots & a_{c_{c}p_{p}} & a_{c_{c}c_{1}} & \cdots & a_{c_{c}c_{c}} \end{array}\right)$$

### 3.2. Modify (Step 2)

To date, all the connections related to subsystems have been laid out in matrix *A*. As mentioned earlier, each nonzero element in matrix *A* shows a connection between corresponding nodes while preserving the direction of the connection. However, a closer look at the indices assigned to each element of matrix *A* reveals that *A* consists of four distinct parts, each one of which denotes a particular type of relationship, as shown in the following equations:

(1) $$Type1:\{a\left(i,j\right)|i\in V_{p},\;j\in V_{p}$$

(2) $$Type2:\{a\left(i,j\right)|i\in V_{c},\;j\in V_{c}$$

(3) $$Type3:\{a\left(i,j\right)|i\in V_{p},\;j\in V_{c}$$

(4) $$Type4:\{a\left(i,j\right)|i\in V_{c},\;j\in V_{p}$$

Equations (1) and (2) point to an intra-dependency, where two nodes from the same subsystem are connected. On the other hand, linking two different types of nodes as in Equations (3) and (4), forms an inter-dependency between two subsystems and, it means that the performance of one node in the host subsystem depends on one node from another subsystem. Although various types of dependency within subsystems are identified in the matrix A, yet its distributed pattern caused these data to remain elusive so far. To address this challenge, we apply a systematic approach based on the general concept of DSM [50] to modify the current structure of nodes in a way that the salient connectivity properties of each node could be identified and utilized in further processing.

We use complex numbers to distinguish between different types of dependencies. All the nonzero elements of Type 2 and Type 4 in matrix *A* turn to imaginary, i.e., 1 is represented as i. Then, all the elements of Type 3 transpose and merge with the elements of Type 4. The new structure, called MDSM, is as follows:

$$MDSM=\left(\begin{array}{cccccc} a_{p_{1}p_{1}} & \cdots & a_{p_{1}p_{p}} & 0 & \cdots & 0\\ : & ... & : & : & ... & :\\ a_{p_{p}p_{1}} & \cdots & a_{p_{p}p_{p}} & 0 & \cdots & 0\\ a_{p_{1}c_{1}}+ia_{c_{1}p_{1}} & \cdots & a_{p_{p}c_{1}}+ia_{c_{1}p_{p}} & ia_{c_{1}c_{1}} & \cdots & ia_{c_{1}c_{c}}\\ : & ... & : & : & ... & :\\ a_{p_{1}c_{c}}+ia_{c_{c}p_{1}} & \cdots & a_{p_{p}c_{c}}+i_{c_{c}p_{p}} & ia_{c_{c}c_{1}} & \cdots & ia_{c_{c}c_{c}} \end{array}\right)$$

Modifying the structure of matrix A provides clear and meaningful insight into the interactions among system components while decreases the complexity. Having the MDSM, one can easily access to different types of dependency in predefined spots that will facilitate further study and computations.

### 3.3. Rearrange (Step 3)

This step aims to represent a compact view of the system interactions by decreasing the distance between nonzero elements of the MDSM while preserving the system topology.

Recent studies discovered that most of the complex systems like CIs have a scale-free characteristic [52]. These systems, particularly the power and communication systems, have less redundant links; this means that the graph representing such systems will be sparse (a graph G=(V,A) is sparse if |A| is much smaller than ${\left|V\right|}^{2}$), and consequently the resulting matrix A for such systems will, in general, be sparse [53]. Table 1 is an example of this sparsity which compares the number of links and nodes in several standard IEEE test systems. Imagine that we want to demonstrate connections among the 118 components of the IEEE 118-Bus (without considering the second subsystem). The adjacency matrix of this system is a 118×118 matrix, in which only 179 elements out of the total 13,924 elements are nonzero. This means that a large number of elements in a 118×118 matrix that are spread in the matrix A should be examined to analyse the connection properties, even though only 1.3% of the elements are nonzero.

MDSM has been designed to facilitate the analysis of connection properties and in particular, the identification of characteristics of dependency in large scale CPSs. To this end, minimization of the distance between nonzero elements of the MDSM will enhance the efficiency of the method, will provide better visualization, and will reduce the computational complexity of mathematical methods that can leverage the MDSM; such minimization can be achieved by appropriately reordering the columns of the MDSM. These columns will be moved with their labels to preserve the system topology. However, reordering the columns of MDSM to decrease the distance between the nonzero elements of one row could increase the distance between the nonzero elements of the other rows. Besides, there might be different permutations that lead to similar, as regards the optimality criterion, results. Thus, an optimization algorithm is required to compute the global optimum for rearranging the MDSM.

Several algorithms have been proposed during the last two decades to solve optimization problems in different domains, such as the Genetic Algorithm (GA) [54], the Simulated Annealing (SA) [55], the Ant Colony Optimization (ACO) [56] and the Imperialist Competitive Algorithm (ICA) [57]. Among them ICA, which was developed based on the swarm intelligence theory by Atashpaz et al. [57], has been widely applied to address different optimization problems in engineering, scheduling, data clustering, network flows, facility layout and neural networks, to name a few [58]. In [58] the superiority of ICA as compared to other evolutionary algorithms, in particular regarding its flexibility, robustness, reasonable computational time, scalability and ability to handle a large number of decision variables was established. These characteristics, as well as the wide range of problems that have been solved by ICA in engineering, make the ICA an ideal choice to apply in the Rearrange step of our proposed method. In the sequel, we describe how ICA can be used to address the MDSM columns reordering problem.

ICA begins with an initial population; each individual of this population is called a country. Then, some of the best countries (with the least cost) are labelled as imperialists, and the rest of them will be the colonies of these imperialists. In our case, each country represents one possible permutation of columns in the MDSM. For each country, the cost is defined as the sum of the absolute distance between every two nonzero adjacent elements in all rows of MDSM. This is computed by means of the Cost Function, the pseudo code of which is shown in Algorithm 1.
**Algorithm 1.** (Cost Function).1:**for**i=1 to $N_{row}$ **do**2:    Find the column index of non-zero elements in row *i* and store in the vector *P*;3:    **for** k=1 to Numel(P)−1 **do**4:        Compute the $CostFunction=P_{k}-P_{k+1}$;5:    **end for**6:**end for** 

Based on the power of the imperialists, which is inversely proportional to the cost, all the colonies are divided among the imperialists and each imperialist together with its colonies form an empire. After that, according to the two main operators, Assimilation and Revolution, colonies start moving toward their relevant imperialist and the Intra-Empire Competition starts. In case the power of a colony exceeds the power of the associated imperialist inside the same empire, that colony and the imperialist swap roles. Then the Inter-Empire Competition begins, in which the weakest empire loses its weakest colony and thereby its power decreases, while the winner of the inter-empire competition will possess that colony and in consequence gain more power. The power of each empire is computed based on a linear combination of the imperialist’ power and the mean power of its relevant colonies in the empire. Through the imperialistic competition, the powerful empires will gradually grow and gain more power. The result of this process identifies the optimum permutation for reordering the columns of MDSM.

Rearranging the columns of a sparse MDSM using ICA will increase the efficiency of further computations, and will provide a better display. Additionally, the proposed method could be also applied to other domains and systems with dense connections. In this case, step 3 (discussed in Section 3.3) could be skipped without affecting the final result.

### 3.4. Display (Step 4)

The ICA will identify the optimum permutation of columns in MDSM in polynomial-time and will show it as a vector of size *p*, where *p* denotes the number of elements in the first subsystem (physical) under study. According to this vector, the columns of MDSM are rearranged and the MDSM is updated. The new structured arrangement of elements and interactions in MDSM provides an appropriate compact representation for complex CPSs. In comparison with previous network modelling approaches such as those utilizing graph and adjacency matrix, MDSM can extract meaningful relations among components of a large scale system and represent it in a predefined and relatively small space.

**Figure 2:**
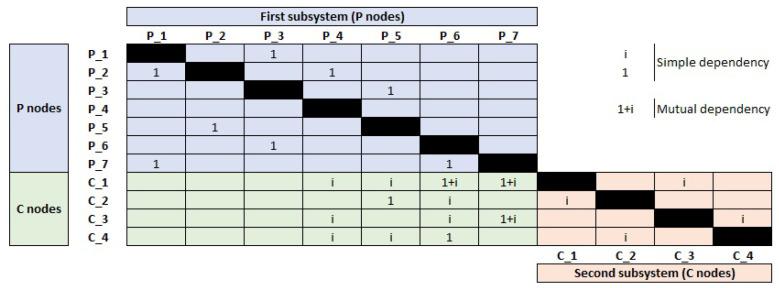
MDSM.

To demonstrate the structure of MDSM a small scale sample of MDSM is presented in Figure 2, which reflects the relationships between two subsystems of order p×c. As illustrated in Figure 2, MDSM categorised connections into three parts. The green part displays the inter-dependency between two subsystems which we call it the “inter-dependency part”, while the blue and the orange parts, named as “intra-dependency part”, refer to the connections inside the first subsystem and the second subsystem, respectively.

### 3.5. Identify (Step 5)

**Figure 3:**
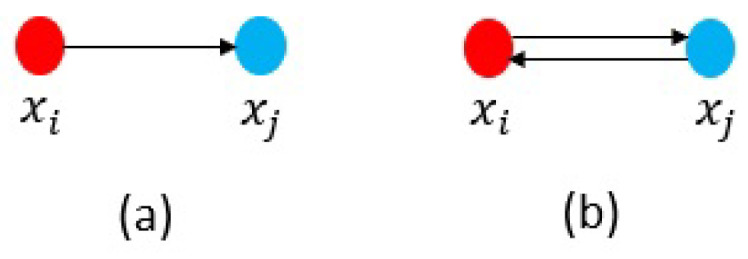
(**a**) Dependency (simple dependency) and (**b**) Interdependency (mutual dependency).

Once the MDSM is displayed, characteristics of a complex system could be simply observed, and relationships among the components become apparent from even a cursory review. The MDSM particularly highlights the dependency patterns that could be divided into dependency (i.e., simple dependency) and interdependency (i.e., mutual dependency) as shown in Figure 3.

**Figure 4:**
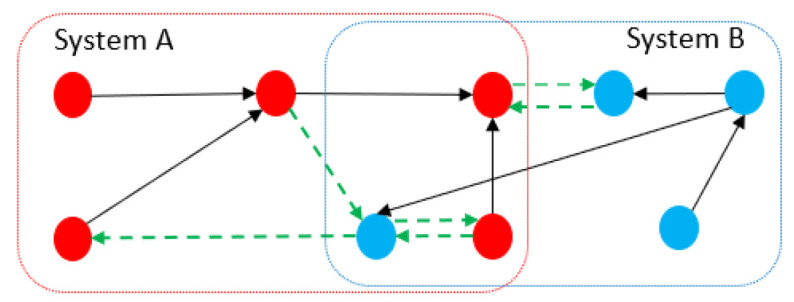
The green dashed edges represent the dependency within a complex system.

Dependency is demonstrated by 1 or *i* in the MDSM and shows that one component in a subsystem depends on another component, either from the same or the other subsystem (Figure 2). However, interdependency is a bidirectional path between two components belong to two different subsystems $V\times V\equiv \{\left(x_{i},x_{j}\right)|x_{i}\in Vp\;\&\;x_{j}\in Vc\}$, and indicates the presence of two paths, i.e., $x_{i}\to x_{j}$ and $x_{j}\to x_{i}$ in the directed graph of the system which means that $x_{i}$ and $x_{j}$ depend on each other. This is displayed as a complex number i+1 in the inter-dependency part of the MDSM in Figure 2 and shows that in a CPS, one can have access from the first subsystem (i.e., physical layer) to the second subsystem (i.e., cyber layer) and vice versa. When two systems are connected, the new compound system could be more fragile than each of its constituents as unforeseen dependencies between two systems can be targeted by attackers, or a simple failure in one part may lead to cascading failures in the entire system. For instance, attackers might leverage a dependency link between two systems as an infiltration point to make an attack path into the other system (see Figure 4). Therefore, dependency and interdependency in the inter-dependency part of an MDSM could be considered as jumping points between subsystems, and analysis of these points could play important role in mitigating risks and enhancing security and safety in CPSs.

### 3.6. Analyze (Step 6)

The identification of different types of dependency, and more precisely in the inter-dependency part of the MDSM, in step 5 provides the essential requirements for quantitative analysis of the criticality and the impact of each dependency between two subsystems. Dependencies between two subsystems could affect the behaviour of a whole CPS as a system-of-systems in different ways and might cause undesired consequences. For these reasons, by utilizing the proposed inter-dependency part of the MDSM we scrutinize these different aspects and develop quantitative parameters to evaluate the effect of dependencies on the operability of the entire system. Considering the parameters proposed in [59], we define four parameters to study the characteristics of the dependent components between two subsystems, namely Impact of Dependency (IoD), Susceptibility of Dependency (SoD), Weight of Dependency (WoD) and Criticality of Dependency (CoD). We also present the concept of the higher order of dependency based on the proposed parameters to evaluate the chain of dependencies in systems. The proposed parameters are defined in the following paragraphs.

#### 3.6.1. Impact of Dependency (IoD)

In the MDSM, IoD determines the impact of one particular node $x_{i}$ on the components of another subsystem under study, by measuring the number of components that are influenced by that node $\left(x_{i}\right)$. $IoD_{Inter}$ shows how many components in a subsystem depend on the functionality of a single node in another subsystem. In other words, it measures the potential power of a node to affect another subsystem.

Based on the MDSM, it is also possible to measure the impact of each node within the system it belongs to with $IoD_{Intra}$. However, our emphasis here is mainly on the analysis of the interactions between two subsystems and corresponding consequences.

To compute the $IoD_{Inter}$ of the *i*th node from the first subsystem (i.e., $p_{i}$), one needs to count how many times the real number “1” is shown in the *i*th column of the inter-dependency part in the MDSM. Equation 5 shows how this parameter is measured.

(5) $$IoD_{Inter}\left(p_{i}\right)=\sum_{j=1}^{c}Re\left(p_{i},c_{j}\right)$$

$IoD_{Intra}$ of the *i*th node from the first subsystem (i.e., $p_{i}$) counts how many times the real number “1” is shown in the *i*th row of the intra-dependency part of the first subsystem in the MDSM (Equation (6)).

(6) $$IoD_{Intra}\left(p_{i}\right)=\sum_{j=1}^{p}Re\left(p_{i},p_{j}\right)$$

For each node of the second subsystem (i.e., $c_{i}$), the values of $IoD_{Inter}\left(c_{i}\right)$ and $IoD_{Intra}\left(c_{i}\right)$ are computed by counting the instances of the imaginary number “i” in the *i*th row of the inter-dependency part and the intra-dependency part of the second subsystem in the MDSM, respectively.

(7) $$IoD_{Inter}\left(c_{i}\right)=\sum_{j=1}^{p}Im\left(c_{i},p_{j}\right)$$

(8) $$IoD_{Intra}\left(c_{i}\right)=\sum_{j=1}^{c}Im\left(c_{i},c_{j}\right)$$

Notice that $\left(p_{i},c_{j}\right)$ indicates the directed path from $p_{i}$ to $c_{j}$, while $\left(c_{i},p_{j}\right)$ refers to the directed path $c_{i}\to p_{j}$. For instance, the $IoD_{Inter}$ of the first element in the second subsystem (i.e., $IoD_{Inter}\left(c_{1}\right))$, in Figure 2 equals to 4, since $c_{1}$ has access to four elements of the first subsystem. Likewise, the value of $IoD_{Inter}$ of $P_{6}$ is equal to 2, i.e., $IoD_{Inter}(p_{6})=2$.

**Figure 5:**
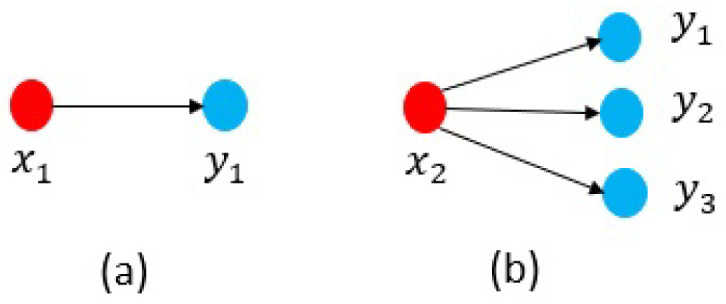
Impact of dependency; (**a**) $IoD_{x_{1}}=1$ and (**b**) $IoD_{x_{2}}=3$.

In general, nodes with a higher value of IoD have more impact on the system. For instance, in Figure 5a, if node $x_{1}$ fails, only one node $y_{1}$ fails too. However, in Figure 5b three nodes $\left\{y_{1},y_{2},y_{3}\right\}$ will stop working, by the $x_{2}$ failure.

#### 3.6.2. Susceptibility of Dependency (SoD)

Susceptibility of dependency shows how much the operability of one node in a subsystem is depending on the operability of other nodes in another subsystem. For each component in a subsystem, the more links a node receives, the higher level of susceptibility it has.

Assume that we are interested in computing the $SoD_{Inter}$ of $p_{j}$ from the first subsystem. According to the inter-dependency part of the MDSM, we simply need to count the number of links incident upon $p_{j}$ from the second subsystem, which is represented by the imaginary number “i” (see Equation (9)).

(9) $$SoD_{Inter}\left(p_{j}\right)=\sum_{i=1}^{c}Im\left(c_{i},p_{j}\right)$$

As shown in Equation (10), $SoD_{Intra}$ of the *j*th node from the first subsystem (i.e., $p_{j}$) counts how many times the real number “1” is shown in the *j*th column of the intra-dependency part of the first subsystem in the MDSM.

(10) $$SoD_{Intra}\left(p_{j}\right)=\sum_{i=1}^{p}Re\left(p_{i},p_{j}\right)$$

Likewise, for those nodes that belong to the second subsystem in the MDSM (i.e., $c_{j}$), the $SoD_{Inter}\left(c_{j}\right)$ and $SoD_{Intra}\left(c_{j}\right)$ is calculated based on the following equations:

(11) $$SoD_{Inter}\left(c_{j}\right)=\sum_{i=1}^{p}Re\left(p_{i},c_{j}\right)$$

(12) $$SoD_{Intra}\left(c_{j}\right)=\sum_{i=1}^{c}Im\left(c_{i},c_{j}\right)$$

As an example, the $SoD_{Inter}$ of $p_{6}$ in Figure 2, is equal to 3, because three links from $\left\{c_{1},c_{2},c_{3}\right\}$ towards $p_{6}$ exist. In line with Equation (11), the value of $SoD_{Inter}\left(c_{1}\right)$ in Figure 2, is equal to 2 (i.e., $SoD_{Inter}(c_{1})=2$).

**Figure 6:**
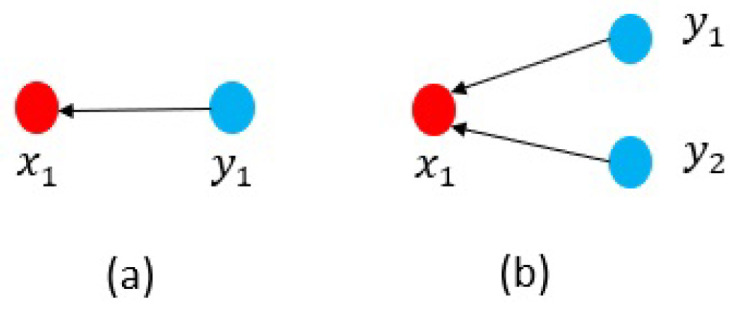
Susceptibility of dependency; (**a**) $SoD_{x_{1}}=1$ and (**b**) $SoD_{x_{1}}=2$.

Indeed, the susceptibility of dependency is a useful parameter from both the defender and attacker point of view. For example, suppose that an attacker tends to target a highly protected node $x_{1}$ in Figure 6. Due to the cost of the attack, the attacker may alternatively attempt to target node $x_{1}$ through the $x_{1}$’s neighbour nodes. In this case, with reference to Figure 6b the attacker could influence node $x_{1}$ either through $y_{1}$ or $y_{2}$. However, in Figure 6a there is only one option. As mentioned earlier, the more links a node receives, the higher level of susceptibility it has. This parameter could be applied to investigate attack surfaces in complex systems as well as to analyse and predict probable attack paths.

#### 3.6.3. Weight of Dependency (WoD)

In general, SoD and IoD measure that how many components in a system are affected by or impacted on other components because of the presence of a dependency in a system. However, the strength of dependency differs with its type. An interdependency in a system has a higher impact compared to a dependency. That is because if a node at one end of an interdependency fails or one of the mutual dependency links stop working, the corresponding node at the other end might not act properly, and the response would not be sent via the other link, i.e., the other link will also fail.

An interdependency has the potential of making common cause failure or even cascading failures to form a closed-loop in the system, which can continuously oscillate the values and states of the connected components. This would be more clear from the security perspective.

Accordingly, the weight of dependency which is assigned to an interdependency is α times greater than that of a dependency. Parameter α is defined as a power of 2, $\alpha =2^{n}$, in which *n* could be adjusted based on the importance of interdependencies for specific purposes and domains, but in general, it is defined as follows:

$$\begin{array}{c} \;(\mathrm{Dependency})\;\;:\;n=0\to \alpha =1\\ \left(\mathrm{Interdependency}\right):\;n=1\to \alpha =2 \end{array}$$

#### 3.6.4. Criticality of Dependency (CoD)

Each system or subsystem consists of several components whose functionality highly affects the performance of the entire system; these are known as the critical components. To enhance the reliability of systems, we always try to keep the critical components away from any failures or unsecured connections and various methods have been proposed to identify critical components. However, once these components are identified within a system, it is still essential to protect them from potential vulnerabilities that might arise as a consequence of connecting new components or subsystems to the main system. It is precisely at this point that MDSM could be of great aid in modelling connections of a system-of-systems, and provide a clear view of these critical components in terms of connectivity. Based on that, Criticality of Dependency (CoD) measures how close a critical component from one subsystem is to components of the other subsystem. The CoD along with other proposed parameters, SoD, IoD and WoD, will help one to study the properties of dependency links in a system-of-systems and investigate whether these connections might threaten the critical components of the system and increase the risk.

In the following examples, we measure the first order of the CoD which shows whether there is a direct connection between a component in one subsystem and a critical component in another subsystem, or not. Imagine that $c_{1}$ is identified as the critical node of the second subsystem in Figure 2. Then, because of the connection between $\left\{p_{6},p_{7}\right\}$ in the first subsystem with $\left\{c_{1}\right\}$, the CoD of these two components are not zero, which means $CoD_{P_{6}}=1$ and $CoD_{p_{7}}=1$.

Depending on the level of the accuracy needed to determine the CoD in a system, it is also possible to consider the value of criticality of components in a system instead of having a binary view. Now for a non-binary example, suppose that $c_{1}$ and $c_{3}$ are both critical nodes of the second subsystem and it has been defined that the role of $c_{1}$ is three times more vital than $c_{3}$. In this case, the $CoD_{p_{6}}$ and $CoD_{p_{7}}$ would be determined as 3 and 4, respectively.

The parameter CoD along with other proposed parameters, SoD, IoD and WoD, will help one to study the properties of dependency links in a system-of-systems and investigate whether these connections might threaten the critical components of the system and increase the risk. In summary, parameters SoD and IoD not only detect the dependent links between subsystems, but also help to evaluate the importance and the impact of those links when compromised, while CoD and WoD describe the properties of each dependency.

## 4. Higher Order of Dependency in System-of-Systems

As discussed earlier, coupling different systems and infrastructures might increase the vulnerability, as one failure in a system could lead to another failure in the other system and this process could continue back and forth until all connected components, and subsystems fail. Recent blackouts in the US [2], and Italy [60] and their severe impacts are concrete examples of such a cross-sectoral cascading failure in the interconnected infrastructures. These power outages and similar crises in recent years have raised many questions regarding the effect of different types of connections, and the impact of systems rewiring in improving the resilience of the interdependent infrastructures.

In Section 3, four parameters were introduced to extract different characteristics of connections in CPSs. Nevertheless, evaluation of the multi-order of dependency in such interconnected systems could provide a more precise picture of interactions, dependencies, and cascading effects. For these reasons, we define the Higher order dependency (HoD) as a parameter to analyse a system not only based on the direct interactions, but also by considering the chain of dependencies, the impact of the structure of systems, and the effect of all the components in complex systems. To further improve the depth of analysis, HoD could be applied along with the other parameters of dependency. To define the concept of higher order dependency we use the terminology of Graph Theory in [61]. In the directed graph *D*, for all integer *p*, $N_{D}^{p}\left(x_{i}\right)$ denotes the pth out-neighbourhood node $x_{i}$. For instance, if node $x_{i}$ has a direct connection with nodes $\left\{x_{j},x_{k}\right\}$, then the first out-neighbourhood $x_{i}$ is defined as $N_{D}^{1}\left(x_{i}\right)=\left\{x_{j},x_{k}\right\}$. Furthermore, if node $x_{j}$ is connected to $x_{l}$, the second out-neighbourhood node $x_{i}$ will be $N_{D}^{2}\left(x_{i}\right)=\left\{x_{l}\right\}$. Indeed, the pth out-neighbourhood of one node represents the pth order of dependency for that node. The higher order of dependency for node $x_{i}$ is determined as follows:

$x_{i}\to x_{k}|\;$ (First Order)

$x_{i}\to x_{j}\to x_{l}|\;$ (Second Order)

where the first order of the chain of dependency for $x_{i}$ includes two nodes $\left\{x_{j},x_{k}\right\}$ and the second order only has one node $\left\{x_{l}\right\}$.

The Breadth-First Search (BFS) is an algorithm that could be applied to extract the higher order of dependency. The BFS explores and extracts all the neighbour nodes of each node in a system. In the worst-case, the time complexity of this algorithm is O(|V+A|) and the required space for saving the result is O(|V|) [61]. Based on the level we wish to explore the order of dependency in a system, the time complexity of applying this algorithm to extract the chain of dependencies varies, but in the worst case will be O(|V+A|). Note that *V* and *A* are the numbers of nodes and links in a system, respectively.

One approach to compute the value of the HoD is to add together the value of each order. In this case, each order of dependency in the chain of dependency with the length *n* has the same impact. However, the effect of dependencies in a system tends to decrease with an increase in distance [26]. This will be further explained with the case study in Section 5.

Kotzanikolaou et al. [26] utilized multi-order dependencies to investigate the effect of disruption to interconnected infrastructures. They proposed an equation to compute the cumulative dependency risk based on likelihood and impact considering the chain of dependency among different systems. Here we modify their equation to compute the nth-order of dependency without considering the concept of risk. Let $Y_{0}\to Y_{1}\to ...\to Y_{n}$ be a chain of dependency of length *n*. Then, according to [26], the nth-order of outgoing dependency of $Y_{0}$, denoted as $D_{Y_{0}}^{n}$, is computed by:

(13) $$D_{Y_{0}}^{n}=\sum_{i=1}^{n}\left(\prod_{j=1}^{i}D_{Y_{j-1},Y_{j}}\right)$$

where $D_{Y_{j-1},Y_{j}}$ is a link between two elements, $Y_{j-1}$ and $Y_{j}$. For example, based on Equation (13), the 3rd-order of outgoing dependency of $Y_{0}$ is computed as: $D_{Y_{0}}^{3}=D_{Y_{0},Y_{1}}+D_{Y_{0},Y_{1}}.D_{Y_{1},Y_{2}}+D_{Y_{0},Y_{1}}.D_{Y_{1},Y_{2}}.D_{Y_{2},Y_{3}}$. Here, the term $D_{Y_{0},Y_{1}}.D_{Y_{1},Y_{2}}$ denotes that $Y_{0}$ is connected to $Y_{2}$ through the two links $D_{Y_{0},Y_{1}}$ and $D_{Y_{1},Y_{2}}$. Therefore, considering the Equation (13), multi-order dependencies for each element comprise *n* times of the first order of dependency, n−1 times of the second order of dependency and so on. For simplicity, we can rewrite Equation (13) as follows:

(14) $$HoD_{x_{i}}=nN_{D}^{1}\left(x_{i}\right)+\left(n-1\right)N_{D}^{2}\left(x_{i}\right)+...+N_{D}^{n}\left(x_{i}\right)$$

Here, *n* defines the order of dependency. Equation (14) can be applied in different cases to measure the risk, impact and susceptibility by considering the chain of dependency. Unlike [26], all the feedback loops between two subsystems are considered in our study as those are part of the system structure. We will apply the higher order dependency and will discuss the result in Section 5.

## 5. Case Study

**Figure 7:**
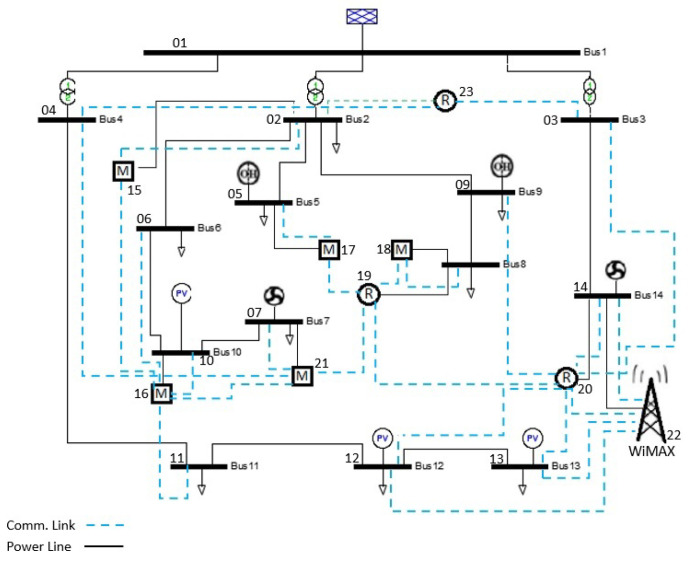
Network structure extracted from a real French distribution network, G2ELAB 14-Bus [33].

In this section, we analyse the proposed dependency parameters based on the micro-distribution network that was developed on the basis of a real French distribution network with 14 power-bus, called G2ELAB 14-Bus. This system includes both Electric Power System (EPS) and the ICT system (see Figure 7), and has been broadly used in related studies [33,62,63]. Although the advantages of MDSM as a graphical model could be recognized better in large scale systems, this system has been chosen for educational purposes and for allowing the comparison of our results with those in previous works.

**Figure 8:**
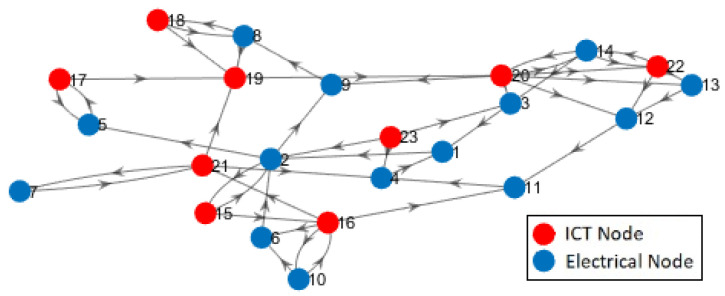
Digraph of the test system.

In the test system, the EPS (first subsystem) includes 14 power buses, 7 distributed generation sources, 17 lines, 9 loads, and 3 transformers HV/MV and the ICT system (second subsystem) consists of 1 Wimax BS, 5 multiplexers, 3 routers [33,62]. For the sake of simplicity, the digraph of this system is shown in Figure 8, where red circles represent the electrical nodes that belong to the physical part (first subsystem) and nodes of the cyber part (second subsystem) are depicted in blue colour.

Sanchez et al. [62] modelled this system as undirected and directed graphs and measured the Betweenness Centrality and Efficiency of nodes for both perspectives to identify the system vulnerabilities. Later, Milanovic et al. [33] followed the same approach and modelled the system as unidirectional and bidirectional graphs to compute the Node Degree and Efficiency of different types of connections (see Equations (1)–(4) in Section 3) by utilizing complex numbers. The authors also proposed a three-dimensional interconnected model to represent the connections between interconnected ICT and EPS. However, to show the interaction between the two interconnected systems, their model needs two separate matrices. Besides, they asserted that owing to assigning different values such as 1, i, and 1 + i to each type of connections in the system, the computational complexity of the method is relatively high. On the contrary, our proposed MDSM can be applied to modelling unidirectional graphs, bidirectional graphs as well as complex systems with hybrid graphs. Moreover, the usage of complex numbers in MDSM is quite different from previous works. In a nutshell, all of those linkages $a=\langle \vec{u,vs.}\rangle$, either inter-dependency or intra-dependency, which originated from the second subsystem are shown with i, while different types of dependency are recognized based on their predefined position in MDSM.

**Figure 9:**
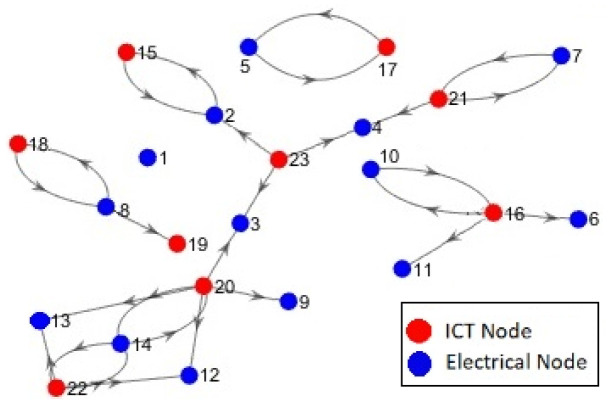
Digraph of the dependency part in MDSM of the test system.

Based on the topological data of the system, we first construct the MDSM and utilize its dependency part to compute the dependency parameters. The digraph of the dependency part is also depicted in Figure 9 to facilitate the understanding of the interdependency and of the closed-loops that exist between the two subsystems.

**Figure 10:**
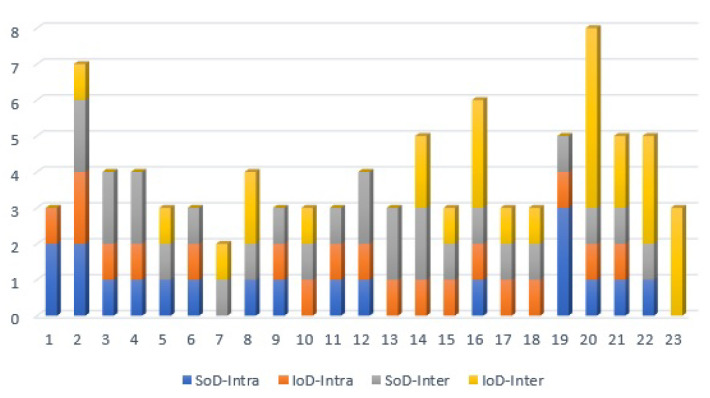
Node degree of the test system: $\left\{SoD_{Inter}+SoD_{Intra}+IoD_{Inter}+IoD_{Intra}\right\}$.

Milanovic et al. [33] argued that the importance of each node in a system can be measured by means of the node degree. Therefore, they computed the node degree of the ICT and EPS components of the test system and concluded that nodes {2,14,16,19,20} are the most important ones. Unlike previous works, the degree distribution of each node in our proposed method is divided into four distinct parts, $\left\{SoD_{Inter},SoD_{Intra},IoD_{Inter},IoD_{Intra}\right\}$, which helps to identify the characteristics of each connection and the role of the corresponding nodes in a system. In our method, the total SoD (i.e., $SoD_{Inter}+SoD_{Intra}$) and the total IoD (i.e., $IoD_{Inter}+IoD_{Intra}$) of each node indicates the total number of its inbound and outbound links. Adding these two parameters, the total SoD and the total IoD is equal to the node degree. Figure 10 shows the node degree of each node in the test system.

**Figure 11:**
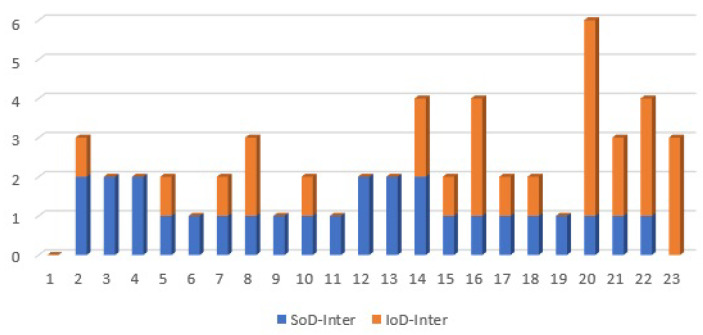
$SoD_{Inter}$ and $IoD_{Inter}$ of the test system.

Nodes 2 and 19 were identified as remarkable nodes in terms of node degree by the authors in [33], which complies with the values shown in Figure 10. However, referring to the values of $\left\{SoD_{Inter},SoD_{Intra},IoD_{Inter},IoD_{Intra}\right\}$ in Figure 10, nodes 2 and 19 are mainly important nodes in their own subsystems, not in the interaction between two subsystems. To make it more clear, Figure 11 depicts $SoD_{Inter}$ and $IoD_{Inter}$ of the test system and reveals that indeed nodes {14,16,20,22} play significant roles in the interaction between two subsystems. In contrast to previous works, our proposed parameters can be applied to distinguish between the attributes of dependencies within a complex system, and between the subsystems of a complex system to identify hidden impacts and vulnerabilities.

The values of $SoD_{Inter}$ and $IoD_{Inter}$ of the test system provide more details of the system connectivity. For instance, for all nodes {5,7,10,14,15,17,18} in Figure 11, the value of $SoD_{Inter}$ is equal to the value of $IoD_{Inter}$. In other words, the number of inbound and outbound links of each of those nodes is the same. This might be a sign of closed-loop/interdependency in the system, as we know that for interdependency between two nodes, if those nodes are isolated, each node has the same number of inbound and outbound links. However, in complex systems, one cannot simply rely on the value of $SoD_{Inter}$ and $IoD_{Inter}$ to identify the interdependencies or closed-loops; one would need more information on the properties of connections.

**Figure 12:**
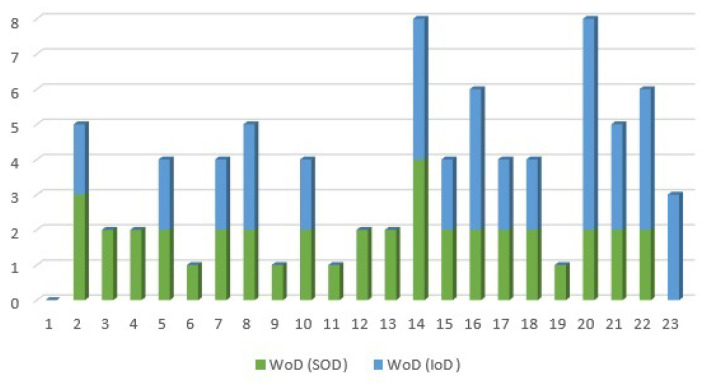
Weight of dependency for SoD and IoD of the test system.

As explained in Section 3, WoD can be applied to measure and to reflect on the properties of dependencies. To this end, the corresponding value of each link based on its type of dependency is taken into account to compute the values of $IoD_{Inter}$ and $SoD_{Inter}$ of each node. The Weight of Dependency of $IoD_{Inter}$ and $SoD_{Inter}$ of the test system is computed and illustrated in Figure 12. Based on Figure 12, measured values of the WoD confirm that each of the nodes {5,7,10,14,15,17,18} is part of a closed-loop. Furthermore, to be more specific, these are the end users in closed-loops, which means that these nodes have no other incoming or outgoing links connected. As an example, based on Figure 11 node 7 has two links, and the WoD of each link in Figure 12 is equal to 2, which clearly shows that node 7 has an interdependency.

Apart from interdependencies, the values of the $SoD_{Inter}$, $IoD_{Inter}$ and the corresponding WoD of each node can be applied to extract the properties of the system connections. For instance, suppose that we wish to analyse the type of dependency of node 22, in the interaction between two subsystems. According to Figure 11, $SoD_{Inter}\left(22\right)=1$ and $IoD_{Inter}\left(22\right)=3$, and Figure 12 shows that $WoD(SoD_{Inter}\left(22\right))=2$ and $WoD(IoD_{Inter}\left(22\right))=4$. Referring to Section 3, we showed that the value of WoD for an interdependency is equal to 2. Here the weight of dependency for one single link $SoD_{Inter}\left(22\right)$ is equal to 2, which confirms that this link is part of a mutual dependency, i.e., an interdependency. For this reason, node 22 has one interdependency that consists of one $IoD_{Inter}$ and one $SoD_{Inter}$, and two dependency links, i.e., $2IoD_{Inter}$ because the WoD of these two links is equal to 2:

$$\begin{array}{cc} \; & SoD_{Inter}\left(22\right)=1,WoD\left(SoD_{Inter}\left(22\right)\right)=2\to \left(1\;Interdependency\right)\\ \; & IoD_{Inter}\left(22\right)=3,WoD\left(IoD_{Inter}\left(22\right)\right)=4\to \left(1\;Interdependency+2\;Dependency\right) \end{array}$$

The results obtained from the analysis of WoD, $IoD_{Inter}$ and $SoD_{Inter}$ are consistent with Figure 9. Therefore, the values of $IoD_{Inter}$, $SoD_{Inter}$, and the corresponding WoD of each node can be used to extract the properties of systems’ connections. These features were not studied in previous works.

**Figure 13:**
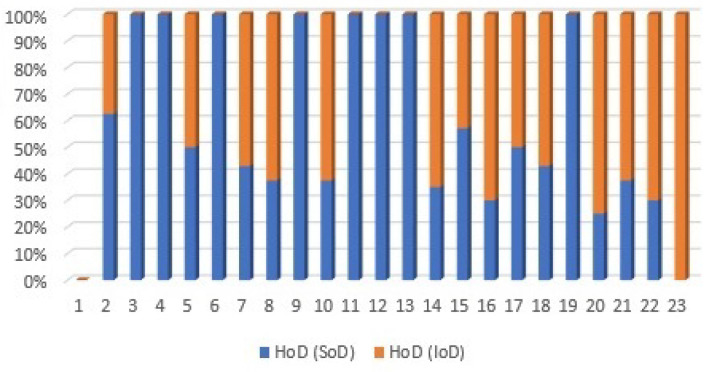
Higher order of dependency for SoD and IoD of the test system.

In Section 3, we also argued that higher order of dependency (HoD) can provide a deeper understanding of interactions between the system components. To evaluate that, the third order of dependency for $SoD_{Inter}$ and $IoD_{Inter}$ of the test system is measured based on Equation (14), in which n = 3; the result is depicted in Figure 13. To date, based on the measured values shown in Figure 11 and Figure 12, we showed that nodes {5,7,10,14,15,17,18} are the end-users of the closed-loops that exist in the test system.

Notably, Figure 13 shows that even in the third order of dependency, the values of the $SoD_{Inter}$ and $IoD_{Inter}$ of nodes {5,17} are still equal. This means that nodes {5,17} form an isolated closed-loop in the system, in which both of these nodes are the end-users.

**Figure 14:**
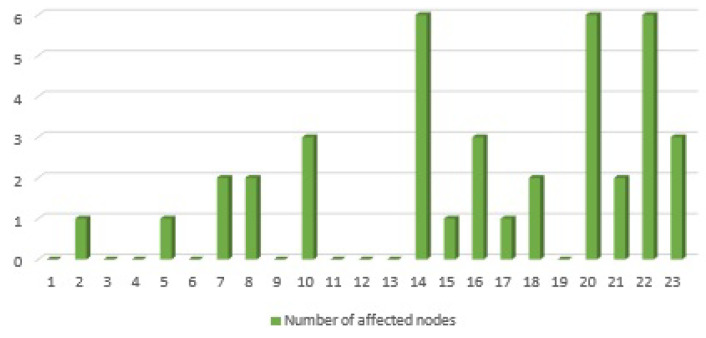
Number of affected nodes by removing each nodes of the test system.

In addition, the nodes {3,4,6,9,11,12,13,19,23} in Figure 11 have either the value of SoD or the value of IoD. Due to the fact that the values of the $IoD_{Inter}$ of nodes {3,4,6,9,11,12,13,19} are equal to zero in Figure 13, these nodes are absolute receiver nodes in the interdependent part of the system. Likewise, given that the value of $SoD_{Inter}\left(23\right)=0$ in Figure 13, node 23 is only a sender. If any of the absolute receiver nodes {3,4,6,9,11,12,13,19} in one subsystem fails, the other subsystem will not be affected (see Figure 9). To make it more clear, we remove each node of the test system and calculate the number of nodes that will be influenced by this removal. The result is depicted in Figure 14. In summary, Figure 14 highlights that removing the nodes with $HoD(IoD_{Inter})=0$ will cause no change in the interdependent part of the system while removing nodes with the higher value of $HoD(IoD_{Inter})$ has a major impact on the connectivity of other nodes.

Taking the higher order of dependency into consideration helps us to better understand the importance of links, and the role of nodes between two subsystems; this is of high value for risk management in complex systems.

The last parameter to investigate on the test system is the Criticality of Dependency (CoD). Based on the betweenness centrality and efficiency, Sanchez et al. [62] stated that nodes {1,2,20,15,19} are vital nodes within this test system. In a follow-up paper [33], the authors expanded the study and introduced {2,5,8,9,16,19,20} as critical nodes based on the Node Degree and weighted Efficiency. Aligned with these papers, a recent study conducted on this system ranked the criticality of each node based on the aggregation of three metrics that measure the importance of each node and its connected links in the entire system [63].

All these recent studies attempt to identify critical components in a complex system, while our purpose here is to determine the critical dependencies between two subsystems. The CoD in a system-of-systems assesses how close one node in a subsystem is to the critical nodes of the other subsystem; this allows us to identify potential vulnerable areas for further investigation. Indeed, once the CoD of a system-of-systems is measured, then we can concentrate on the analysis of other features such as the susceptibility or the impact of those dependencies that have a higher value of CoD in the system, and consequently take proper action to control the consequences, and reduce the risk based on that information.

**Figure 15:**
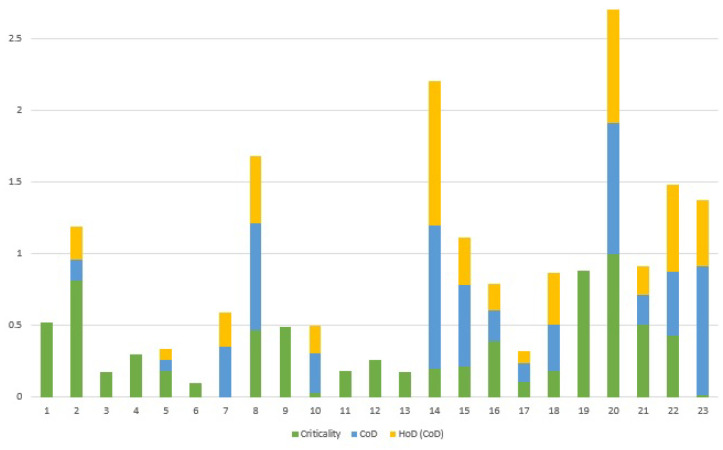
Criticality of nodes, CoD and higher order of dependency for CoD of the test system.

To compute the Criticality of Dependency (CoD) of the test system, we utilize the ranking presented in [63], as it covers all the nodes of the system. Figure 15 displays the criticality of each node, the CoD, and the third order of dependency for the CoD.

Regarding Figure 15, identified critical components in a system are not necessarily those components that have the main role in connecting two subsystems. It should be noticed that when two well-designed and secure systems are merged, the outcome is a system-of-systems in which even less important components of each subsystem might turn to critical components, because of the new linkages. For example, in Figure 15 although node 14 has been not identified as a highly critical node in the test system, its value of CoD indicates that node 14 has a close connection with critical nodes of the system. According to Figure 7, node 14, which is a bus in the first subsystem, is connected to node 20, the main ICT router and the most critical node in the second subsystem. Likewise, {8,23} are two other nodes with the noticeable value of CoD in Figure 15, which are connected to the critical nodes 19 and 8 (from the other subsystem), respectively. As depicted in Figure 9, apart from the interdependency between nodes 14 and 20, these nodes along with nodes {12,13,22} form a local loop; this implies the existence of a vulnerable zone in the system-of-systems. In case that an event adversely affects the functionality of a node and a higher order of dependency turns back to that node, a feedback effect forms in the system which will influence other nodes as well and will exacerbate the total impact of the initial event. Analysis of the higher order of CoD in systems helps us to identify these vulnerable local loops.

The chain of dependency for node 14 shows a direct connection between node 14 (parent) and nodes {20,22} (children) as the first order of dependency, i.e., 14→[20,22]. The second order includes the connections of the children of node 14 which are 20→[3,9,12,13,14] and 22→[12,13,14]. Among the children of the second order only node 14 has further linkages. Therefore, the third order contains the connection between node 14 (as a child in the second order of dependency) and {20,22}. The chain of dependency for node 14 is as follows:

14→20→3×|14→20→9×|14→20→12×|14→20→13×|14→20→14→20|14→20→14→22|14→22→12×|14→22→13×|14→22→14→20|14→22→14→22|

The desired length for extracting the chain of dependency could be adjusted depending on the scale of the system.

In addition to the test system discussed in this section, we developed several test systems with different, large numbers of nodes, in order to evaluate the scalability of the proposed method. All the tests were performed using Matlab R2020a with an Intel Core i7 2.11 GHz processor with 16 GB RAM. To ensure the accuracy of the result, each test was iterated 20 times and both average time and the maximum time recorded. Table 2 demonstrates the outcomes of this analysis. The run times reported in Table 2 show that MDSM can be effectively used to extract the characteristics of dependencies in large scale Cyber–Physical systems.

## 6. Application of Dependency Analysis

Developing a simple model to characterize the structural properties of CPSs such as the interdependencies between subsystems, is of paramount importance to understand and predict the behaviour of systems. What is more interesting is that such a model can be used to extract the chains of influence across multiple subsystems, thereby assisting the vulnerability analysis [64]. As mentioned earlier, MDSM is an intuitive method that can be used for modelling different types of system architectures to display the connections between subsystems within one complex system or the interactions between two critical infrastructures.

MDSM enables system designers and decision-makers to analyse the characteristics of connections inside CPSs to extract dependencies and interdependencies within these systems, to examine a variety of hypothetical scenarios and to anticipate different types of failures that might expand through these links across the entire system.

Dependency parameters of MDSM provide a valuable perspective on the impact of interdependency between subsystems and show how the failure of one subsystem has a domino effect on the others. MDSM provides deep insights into the behaviour of complex CPSs and contributes to system design and recovery as well as to the identification of potential failures and vulnerabilities, security enhancement strategies and risk mitigation. In short, extracting dependency relations in CPSs contributes to satisfying the following objectives:

- Identification of hidden vulnerable zones and dependencies among subsystems.
- Investigation of cascading failures based on the dependency chain inside the systems.
- Analysing the severity and impact of probable failures.
- System design modification in order to mitigate dependencies and consequent failures.
- Developing the system recovery and protection strategies.
- Enhancing the resilience of complex systems.
- Improvement of system security and safety.

## 7. Conclusions

In this paper, we proposed the Modified Dependency Structure Matrix (MDSM) to identify, demonstrate and analyse the characteristics of connections in large scale Cyber Physical Systems. MDSM is a graphical method which aims to provide a compact perspective of inter-dependencies and intra-dependencies that exist between subsystems of a complex system. The dependency parameters introduced in this method, namely Impact of Dependency (IoD), Susceptibility of Dependency (SoD), Weight of Dependency (WoD) and Criticality of Dependency (CoD) provide quantitative measures to determine the characteristics of dependencies among components and subsystems. Unlike previous works, by applying the concept of chain of dependency, MDSM can evaluate the role of each connection in the higher order dependencies and provide a comprehensive perspective regarding the importance of each connection. In general, dependency parameters help to design more reliable and secure complex systems by protecting the critical nodes of each subsystem from vulnerable nodes of the other subsystems and manage this distance by utilizing the chain of dependency at the desired level. The quantitative value of the dependency parameters provides a better view for system designers to modify the architecture of systems, and it helps decision-makers to enhance the security of the system by allocating the budget to more vulnerable zones. As discussed in Section 6, the possible applications of MDSM both as a graphical model and for acquiring dependency parameters are quite many. Among possible options, in future works, we will mainly focus on improving risk management methods as well as developing an attack path analysis model based on the interdependency analysis of CPSs.

## Figures and Tables

**Table 1:** Number of links and nodes in IEEE test systems.

System	N.Nodes	N.Links
IEEE 9-Bus	9	9
IEEE 14-Bus	14	20
IEEE 24-Bus	24	34
IEEE 39-Bus	39	46
IEEE 118-Bus	118	179

**Table 2:** Running time for computing dependency parameters considering the third order of dependency.

Number of Nodes	Avg. Time (per Second)	Max. Time (per Second)
50	0.0049308	0.016134
100	0.0224031	0.10466
500	0.10141165	0.187021
1000	0.40492025	0.440655
5000	29.62836675	30.875652
